# From initial treatment design to final disposal of chelating agents: a review of corrosion and degradation mechanisms

**DOI:** 10.1039/d1ra07272b

**Published:** 2022-01-12

**Authors:** Tariq Almubarak, Jun Hong Ng, Raja Ramanathan, Hisham A. Nasr-El-Din

**Affiliations:** Texas A&M University USA Tariq.mubarak@hotmail.com

## Abstract

The use of aminopolycarboxylic acids (APCAs) is increasing rapidly in several industries because of their unique properties of chelation and their effectiveness in high-temperature conditions. One of the major design considerations before their application is their thermal stability and their corrosivity to tubulars, especially the ones used in the oil and gas industry. Their disposal is also an active topic of discussion. The coordination bond formed between the chelator and metal ions is strong and thus can have long-lasting effects on the environment in terms of the metal's bioavailability. Therefore, its biodegradation and photodegradation must be considered. There is a lack of a single source of these major decision criteria for the selection of suitable APCAs and this paper provides an outlet for researchers and industry professionals to further their understanding of APCAs. Several types of APCAs including EDTA, DTPA, HEDTA, GLDA, NTA, MGDA, CDTA, HEIDA, EDDS, and ASDA were reviewed for their corrosion mechanisms and corrosion rates to the most common tubulars used in the oil and gas industry. In some cases, these chelating agents were implemented as corrosion inhibitors as well. The degradation of APCA was divided into three major categories: thermal-, bio-, and photo-degradation. The influence of temperature, microorganisms, and light play an important role during and post-treatment. To fully understand these degradation mechanisms, literature from several industries including medical, mining, toxicology, hydrometallurgy, materials, environmental sciences, mineral sciences, and electrochemical sciences was examined and elucidated. This paper provides a unique perspective of design considerations with the application of the frequently used APCAs. This review connects literature from several industries and can provide an important step-change in the overall understanding of APCAs from the initial design phase to their final disposal and treatment.

## Abstract 2

In this review, we discuss how chelating agents and their products can cause corrosion and how it goes through the oilfield cycle including thermal, photo, and biodegradation.

## Graphical abstract

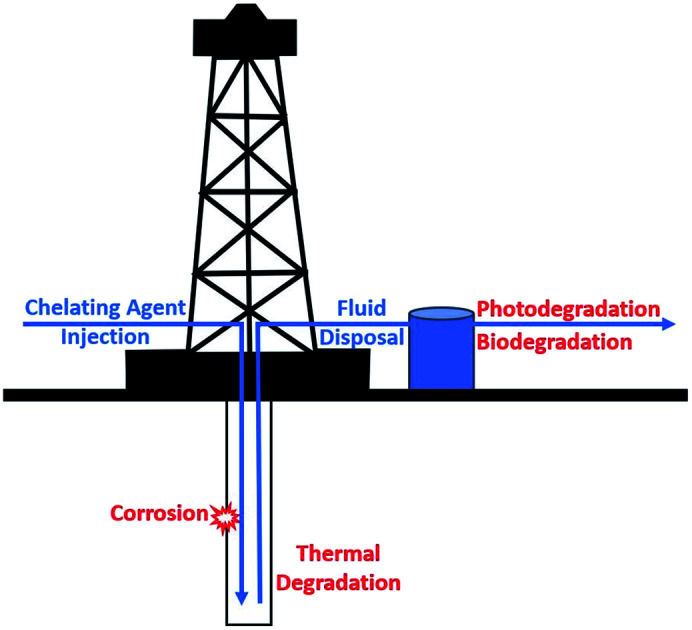

## Introduction

### Chelating agents

Chelating agents are multidentate organic molecules that can form two or more coordination bonds with a central metal ion. The formation of these coordinate bonds involves the donation of electrons from functional groups of the chelating agent to the ion. In doing so, heterocyclic rings known as chelate rings are formed. An important function of chelating agents is to bind with metal ions to form stable complexes, which facilitate the isolation, removal, and transport of these ions. This is especially important to hinder any undesired side reactions involving these ions such as precipitation. In the oil and gas industry, chelators have been used in several applications such as iron control, scale removal, acidizing treatments, and enhanced oil recovery. These applications require chelating agents because of their unique chemistry and mechanism of interactions with the rock, crude oil, and brine components.

Thermodynamic stability between chelators and specific metal ions can be experimentally determined and is referred to as the “stability constant”. It defines the affinity of the chelating agent to the metal ion and plays a role in several practical applications such as metal ion removal, bioavailability, medical treatment, ion exchange, solvent extraction, phase-transfer catalysis, and fuel reprocessing. These constants have been the focus of research for decades and can be found in several comprehensive reference books such as *Critical Stability Constants* by Martell and Smith.^1–7^ Additionally, studies of chelating agents or ligands have shown that the metal–ligand complex is dependent on the size of the ring formed during chelation, the number of rings formed, the basicity of the chelating agent, the nature of the donor metal, and the central metal ion.^8^ A review of the chemistry and dissolution mechanisms of chelating agents has been covered in a previous paper and will therefore only be touched on briefly in this paper.^9^

In the oil and gas industry, chelating agents are used in a variety of applications such as iron control, scale removal, and acidizing. Iron(iii) ions are present during many stimulation treatments due to the corrosion of tubulars by acids or the presence of minerals such as hematite in the formation that react with the stimulation fluid. These ions can cause severe formation damage through the generation of sludges with the formation oil. They can also cause damage through the precipitation of organic molecules present in the treatment fluid.

Chelating agents are also often used to remove a variety of scales such as carbonates, sulfates, and some types of sulfide scale. This is because chelating agents can remove acid-insoluble scales such as barium sulfate that an HCl-based treatment would not be able to resolve. In addition, chelating agents have low corrosivity and do not form many side products from the reaction process compared to the alternative treatments of hydrochloric acid (HCl).

As for acidizing, chelating agents are used primarily for high-temperature treatments where the typical mineral acid or organic acid treatments are unsuitable. This is because of the extreme corrosivity of these acids at these high temperatures and their failure to create a desirable near-wellbore reaction.

Due to the variety of applications at high temperatures, it is important to understand the temperature limits to apply the proper chelating agent within this range. Additionally, while chelating agents are less corrosive than the other treatments, it is critical to note that they are still corrosive and as a result, adequate corrosion inhibition techniques should be implemented with the treatment, as shown in this paper. Lastly, with the ongoing emphasis on cleaner green solutions, it is critical to know how to properly dispose of the chelating agents that were used in treatments to prevent damage to the ecosystem surrounding us.

### Types of aminopolycarboxylic acids (APCAs)

As the name implies, this subgroup of chelating agents contains one or more nitrogen atoms as well as multiple carboxylic acid functional groups. The nitrogen group is typically located at the center of the molecule while the carboxylic acid groups can be likened to “arms” of the chelating agent and bind to ions by “grabbing” them from the solution. This process of “grabbing” is known as chelation and results in the formation of a stable complex that isolates the ion from further reactions.

**Fig. 1:**
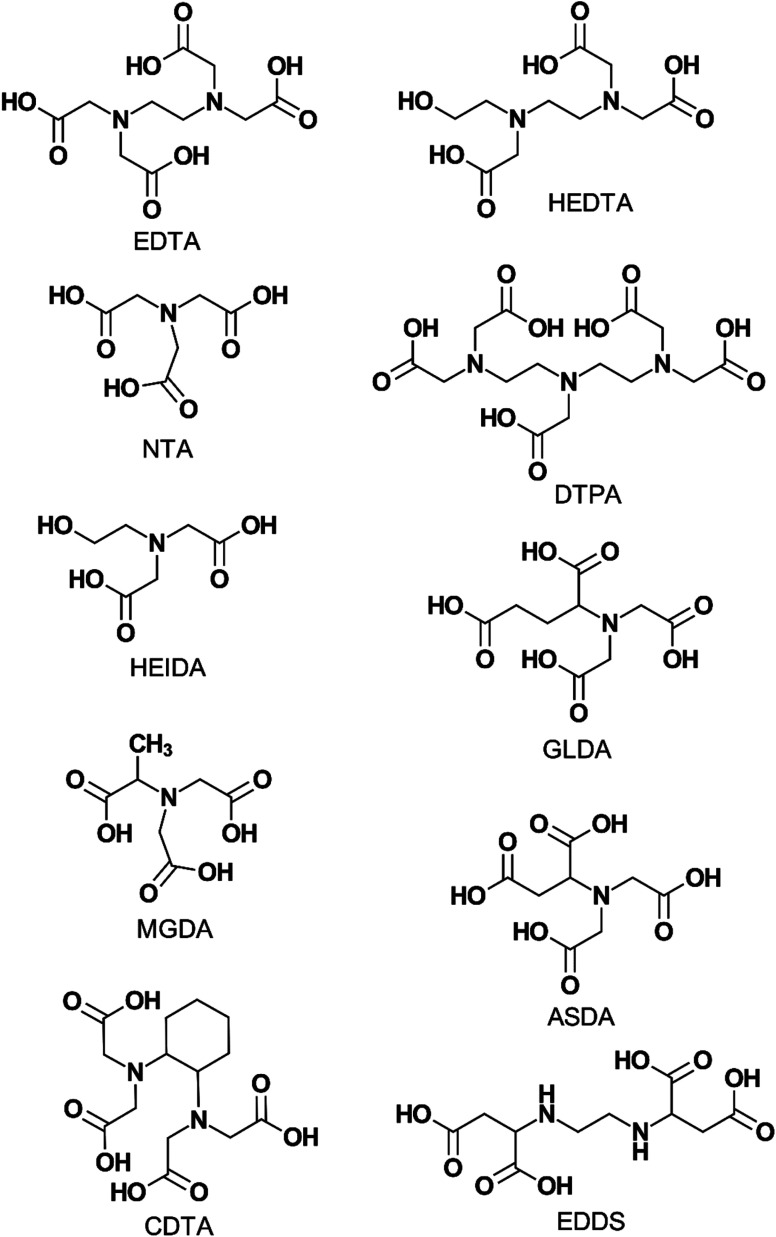
Chemical structures of commonly used aminopolycarboxylic acids.

Fig. 1 shows the chemical structures of the chelating agents mentioned in this review. They are also listed below:

#### 1. EDTA (ethylenediaminetetraacetic acid)

EDTA is a hexadentate aminopolycarboxylic acid that has been used in a variety of applications ranging from detergents to textiles.^10^ In the oil and gas industry, it has also seen a wide variety of applications ranging from stimulation to iron control. However, despite its wide range of applications and common use, EDTA presents several problems. Firstly, it is not readily biodegradable. This, along with its high chelating strength, earned it strict scrutiny in Europe in the late 1980s.^11^ Its use is prohibited in some countries due to its slow biodegradability.^12^ Plant metal uptake increased by 96.8 times in presence of 10 mmol kg^−1^ of EDTA in soil. EDTA inhibits cellular division, chlorophyll synthesis, and algal biomass production.^10^ It has low solubility in acid solutions due to its ampholytic nature.^1^ These disadvantages have spurred researchers to search for alternative chelating agents.

#### 2. HEDTA (hydroxyethylethylenediaminetriacetic acid)

HEDTA is a pentadentate ligand similar in structure to EDTA, with the only difference being that it has a hydroxyethyl group in place of one acetate group. HEDTA was suggested by Frenier *et al.*^13,14^ to replace EDTA as a stimulation fluid. This was due to the low solubility of EDTA at low pH because of its chemical structure. The presence of the hydroxyethyl group in HEDTA instead of an acetate group in EDTA improves the solubility of HEDTA but lowers the stability constant of its metal-complex products. HEDTA has also been used for iron control as well as scale removal.^15–18^ However, it faces similar biodegradability issues as EDTA due to the presence of two nitrogen atoms in its structure.

#### 3. l-GLDA (l-glutamic acid *N*,*N*-diacetic acid)

l-GLDA, often referred to as GLDA, is a relatively new, pentadentate chelating agent.^19^ It is used for iron control as well as stimulation of carbonate and sandstone reservoirs. GLDA has a high solubility in both water and highly concentrated acid solutions.^20^ As a result of its spatial regularity in its chemical structure, GLDA does not crystallize easily and can be more soluble in aqueous solutions. This is due to lowered crystallization tendency.^21^ Furthermore, it is readily biodegradable as it is manufactured from l-glutamic acid or monosodium glutamate. In terms of stability constants, those of GLDA have been generally found to be lower than that of EDTA and HEDTA and are dependent on pH, temperature, and type of metal ions.^22,23^

#### 4. DTPA (diethylenetriaminepentaacetic acid)

DTPA is an octadentate ligand that also has the highest stability constants among commonly used chelating agents in the petroleum industry. However, DTPA is not readily biodegradable^24–26^ and has solubility issues in water and acid solutions. Its most common application in the industry is barium and strontium sulfate scale removal.^27^ A review of APCAs in scale removal applications related to the oil and gas industry is provided elsewhere.^28^

#### 5. NTA (nitrilotriacetic acid)

NTA is a tetradentate aminopolycarboxylic acid that is used for well stimulation, iron control, and scale removal. Its structure consists of three acetic acid “arms” and a central nitrogen atom, all of which are responsible for the denticity of NTA. Although NTA is more biodegradable than other commonly used ligands such as EDTA and HEDTA, it has low stability constant with most cations. In addition, it is a known animal carcinogen and is a restricted chemical in countries such as those in the EU.^12^

#### 6. MGDA (methylglycinediacetic acid)

MGDA is a biodegradable tetradentate chelating agent that was developed based on IDA.^29^ MGDA is prepared by reacting glycine with formaldehyde and alkali metal cyanide in an alkaline medium.^30^ The advantage of MGDA over other ligands is its ability to degrade in the absence of adapted bacteria at standard conditions and to retain stability despite pH and temperature effects.^31^ It is commonly found in cleaning agents such as detergents and dishwashing liquids.

#### 7. CDTA (*trans*-l,2-cyclohexylenediaminetetraacetic acid)

CDTA is a non-biodegradable hexadentate chelating agent that is also commonly used in the medical industry. In the petroleum industry, it has been tested as an alternative acidizing fluid for carbonate formations.^32^ Due to the cyclohexyl group, CDTA is lipophilic in addition to being hydrophilic. This property makes it more effective at alleviating nickel-induced alterations in the body than other chelating agents that are only hydrophilic.^33^

#### 8. HEIDA (hydroxyethyliminodiacetic acid) or HIDA

HEIDA is a tridentate chelating agent with a structure like NTA except it has only two acetate groups and a hydroxyethyl group. It has been used for a variety of purposes including scale removal and acidizing.^17,34^ The advantages of HEIDA are its biodegradability and its solubility, which make it a possible candidate for replacing EDTA. HEIDA is also one of the main thermal degradation products of EDTA.^35^

#### 9. EDDS (ethylenediamine-*N*,*N*′-disuccinic acid)

EDDS is a structural isomer of EDTA. Unlike NTA, EDDS has a high capacity for complexing cations while displaying low toxicity to fish.^36^ In addition, despite being structural isomers, the [*S*,*S*] conformation of EDDS is more biodegradable than EDTA. However, most metal complexes of EDDS are non-biodegradable.^37,38^

#### 10. ASDA (l-aspartic acid *N*,*N*-diacetic acid)

ASDA, along with GLDA, MGDA, HEIDA, EDDS, and several other chelating agents, is a new generation, pentadentate chemical developed in response to a growing need for biodegradable chelating agents to replace non-biodegradable ones such as EDTA and DTPA.^36^ Due to its biodegradability, it has been proposed as a viable substitute to EDTA for cleaning soils poisoned by heavy metals such as copper(ii) (Cu^2+^) and lead(ii) (Pb^2+^) ions.^39,40^

### Types of metals used in the oilfield

In the oil and gas industry, metals of construction can be divided into two distinct categories: low carbon steels (LCS) and corrosion-resistant alloys (CRAs). LCS such as N-80, H-40, or J-55 grade steels are often used to cast tubulars, casings, or pipes that are used downhole or to transport fluids around the facility. CRAs such as 13Cr, S13Cr, and 316L, and nickel-based CRAs such as Hastelloy, Incoloy, and Alloy C-276, are commonly used in environments too corrosive for LCS and are some of the more widely used CRAs in the oil and gas industry.^41^

As implied by their name, carbon steels are a mix between carbon and steel, with the prefix ‘low’ or ‘high’ referring to the percentage composition range of carbon contained in the mixture. Low carbon steels contain less than 0.4%, medium carbon steels contain 0.4% to 0.6%, and high carbon steels contain 0.6% to 1.5% carbon. LCS is the preferred choice of material when downhole conditions are determined to be less corrosive. This is due to their low-cost relative to other steels such as chrome steel, ease of manufacture, and their ability to withstand the physical stresses of downhole conditions. Low carbon steels are relatively cheap and are thus a popular choice for casing and tubing material. If higher yield strength is required, LCS alloys such as P-110 can be used.

CRAs refer to metals such as stainless steel and other non-iron-based alloys such as Hastelloy or Incoloy. These metals usually contain chrome, nickel, and molybdenum to enhance their corrosion resistance. They are often used in formations containing corrosive gases such as H_2_S, CO_2,_ or a combination of both. Both H_2_S and CO_2_ form acidic solutions when dissolved in aqueous media while H_2_S presents the additional problem of Sulfide Stress Cracking (SSC). To control corrosion from these gases, casings, and tubulars made of CRAs are often used in place of LCS. Due to the broad definition of the word corrosion, it must be understood that CRAs are not impervious to all forms of corrosion. Instead, they are metals that display high levels of corrosion resistance specifically in the environment they are in without requiring either inhibition or mitigation techniques. CRAs typically form a layer of Cr_2_O_3_ in the presence of air which confers superior resistance to CO_2_ corrosion. However, concentrated HCl can dissolve this layer, resulting in severe corrosion to the base metal.^42^

### Corrosion

Corrosion is a process that results in the loss of metals through interactions between the metal and the environment around it. In the oil and gas industry, corrosion of downhole equipment and tubulars can result in leaks, equipment failure, or loss of structural integrity. These outcomes can negatively affect production, pose hazards to employees, and incur serious maintenance costs. Costs due to corrosion in the oil and gas industry amount to approximately $1.372 billion annually with more than half the amount resulting from damaged surface equipment and expenses on downhole tubulars.^43^

Acidizing and scale-removal fluids will inadvertently corrode the metal tubulars and damage downhole equipment. Corrosion rates vary due to several factors, including the environmental temperature, type of metal, and treatment duration. Therefore, adequate studies must be conducted beforehand to determine the type and concentration of corrosion inhibitors added to the treatment fluid.

### Types of corrosion

Corrosion can be classified into the following eight different types:^44^ uniform corrosion, galvanic corrosion, crevice corrosion, pitting, intergranular corrosion, selective leaching, erosion, and stress-corrosion cracking. Each corrosion classification involves a different mechanism of attack, and thus requires a different method of prevention. In the petroleum industry, the presence of strong acids and chloride ions causes uniform corrosion, crevice corrosion, and pitting, which are the most prevalent forms.^45^ Under specific conditions, other forms of corrosion have also been observed in the oil field.

**Fig. 2:**
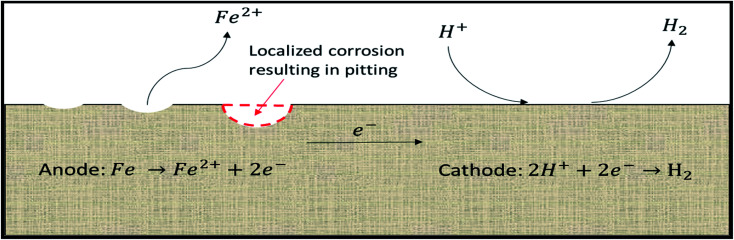
Electrochemical corrosion of iron by acid.

Uniform corrosion is an electrochemical process between a material and its environment that destroys the material. For corrosion to occur, an electrochemical cell consisting of a cathode, an anode, an aqueous medium, and a metallic/electronic path is required. In acidizing, the acid is the aqueous medium and the body of the metal acts as the electronic path through which electrons flow. Due to factors such as grain structure, alloying, and temperature, a metal surface can possess multiple anodic and cathodic sites despite being a single piece of metal. Dissolution of the metal occurs at the anodic sites, while the cathodic reactions range from proton (H^+^) attacks to reduction of water, depending on the environment and the composition of the aqueous solution. Continuous removal of metal from the same anodic site results in pitting and appears as depressions or holes in the metal surface (Fig. 2).

Corrosion from strong acids, such as HCl, on metals, can be primarily described by an electrochemical process. Initially, the acid dissolves the oxide layer of the metal, either iron(iii) oxide or chromium(iii) oxide depending on the type of metal dissolved. This reaction proceeds as shown in eqn (1), where M represents any metal atom:

1 M_*x*_O_*y*_ + 2*y*HCl → *x*MCl_2*y*/*x*_ + *y*H_2_O

The oxide layer on the surface of the metal is generated by the oxidation of the base metal in the air. This layer protects the bulk of the metal from exposure to the environment. Layer thickness is dependent on environmental factors and the structural properties of the metal. After the oxide layer removal, the electrochemical reaction between the bulk metal and H^+^ ions in the solution occurs. Due to imperfections in the metal, local cathodic and anodic sites develop on the metal surface that facilitates corrosion. At cathodic sites, H^+^ ions are reduced through the donation of a pair of electrons from base metal iron (Fe^0^) atoms at the anodic site, as shown in eqn (2).

2 2H^+^ + Fe^0^ → H_2_ + Fe^2+^

Certain compounds present in the metallic structure will affect the corrosion rate of the metal. Cementite, for example, is a form of iron carbide that has been shown to accelerate corrosion by providing a favorable cathodic site with a lower overpotential for the formation of hydrogen.^46^

## Aminopolycarboxylic acids (APCAs)

### Corrosion mechanism

The corrosiveness of various APCAs on metals has been widely studied as they are usually used as alternatives to the traditional HCl acidizing formulations at high temperatures.^47^ APCAs are often used at pH 4 for standalone acidizing. Therefore, understanding chelating agent corrosion is vital when designing the treatment.

**Fig. 3:**
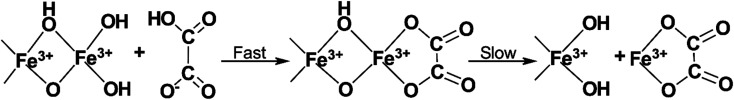
An example of corrosion of the iron oxide layer due to surface complexation by a simple chelating agent (adapted from ref. 48).

The mechanism of dissolution of the metal oxide layer is likely to be similar to that of mineral surfaces.^48^ Ligands adsorb onto the oxide layer of metals and dissolve it through a surface complexation mechanism (Fig. 3). Lewis basic groups on the chelating agent can labilize the central metal ion through the donation of their electron density. This sharing of electron density causes the weakening of other bonds to the metal ion, eventually allowing it to be extracted from the oxide layer. In the case of carbon steel corrosion, accelerated corrosion of the oxide layer may occur due to reductive dissolution.

Reductive dissolution is a well-documented process studied by several authors researching chelate-assisted dissolution of iron-containing minerals such as goethite, magnetite, and hematite.^49–53^ It involves the acceleration of iron oxide dissolution in the presence of reducing agents in the solution. More specifically, the mechanism of reductive dissolution first involves a reducing agent reducing Fe^3+^ in the solution yielding Fe^2+^. Chelated iron(ii) (Fe^2+^–L) then reacts with chelated iron(iii) adsorbed on the surface of the metal (Fe^3+^–L_(ads)_) reducing it in a redox reaction shown in eqn (3). By reducing the charge of the chelated surface iron cation, the dissolution of the chelated ion is improved.

3 Fe^3+^–L_(ads)_ + Fe^2+^–L → Fe^2+^–L_(ads)_ + Fe^3+^–L.

Depending on metal structure and under the right conditions, it is also possible that APCAs enhance corrosion by undergoing reduction at cathodic sites on steel surfaces. Palmer and Boden^54^ showed that the carboxylic acid groups of EDTA were reduced to aldehyde groups when exposed to platinum or mild steel and suggested that EDTA may act as a cathodic stimulant for corrosion of mild steel. Additionally, Calderon *et al.*^55^ observed a higher cathodic slope than that of the cathodic reduction of H^+^ while comparing the corrosivity of disodium EDTA and tetrasodium EDTA on P-110 steel and attributed this observation to EDTA reduction. Ng *et al.*^56^ showed that the chelating agents at pH 4 corrode through a 2-step process: chelator enhanced dissolution of the iron oxide layer followed by a redox reaction between the base metal and the chelating agent. This involves the reduction of the chelating agent carboxylic groups into aldehydes while the base metal Fe is oxidized to Fe^2+^. At low pH, however, the mechanism becomes less clear since, at these conditions, the presence of H^+^ ions in the solution and the likelihood of acid corrosion arises.

Corrosion rates in aggressive environments can be inhibited using a low concentration of chemical compounds called corrosion inhibitors. They act on the metal's surface through different modes such as chemisorption, base-metal oxidation, and reaction with corrosive components. When applied, it protects the metal against pitting, loss of material, the extent of hydrogen embrittlement, and reduction of acid fumes. Corrosion inhibitors are used in acidic, alkali, and neutral environments. Some examples of organic and inorganic corrosion inhibitors include orthophosphates, silicates, chromates, amines, aldehydes, alkaloids, nitro, and nitroso, thiourea, phenols, naphthol, and chelating compounds. The selection of the best corrosion inhibitor is dependent on the type of corrosion, desired protection time, and temperature conditions. A review of several types of corrosion inhibitor packages revealed the wealth of work done in this area for hydrochloric acid-based corrosion of carbon steels.^57^ Chen and Yang discussed the various types of inhibitors used for hydrochloric acid, sulfuric acid, nitric acid, phosphoric acid, hydrofluoric acid, citric acid, and sulfamic acid.^58^ They listed the best-recommended chemistries for the different acid systems used in the oil and gas, metallurgical, mechanical, electrical power, and transportation industries. The corrosion inhibitor packages are specifically designed for each type of acid,^59^ and they vary due to the acid's mechanism of attack on the metal's surface. This review presents a collection of corrosion data relating to the application of aminopolycarboxylic acids in different industries and how the corrosion rates were inhibited.

### Laboratory testing of corrosion rates

Many tests studying the corrosion rates of chelating agents at low pH conditions have been carried out with carbon steels and corrosion-resistant alloys. In general, the corrosion rates are considered acceptable if they are kept below 45.4 mmpy (0.05 lb ft^−2^ over 6 h).^42,60^ For chrome steel, this corrosion rate limit is lowered to 27.2 mmpy (0.03 lb ft^−2^) and for coiled tubing, the corrosion rate limit is 18.2 mmpy (0.02 lb ft^−2^).^42,60^ Corrosion rate depends on the APCA and the type of metal corroded. For example, at high temperatures, acidic formulations (pH 4) of HEDTA were found to require corrosion inhibitors when used with carbon steel tubulars, while alkaline formulations of HEDTA and EDTA did not.^61^ Additionally, acidic formulations of GLDA were shown to require no corrosion inhibitor below temperatures of 149 °C (300 °F) on chrome steel.^62^

Frenier *et al.*^14^ tested the corrosion rate for 2.8% HEIDA in 28% HCl on N80, 13Cr, and CT carbon steels at 149 °C (300 °F). It required a 0.8% HCl acid inhibitor to maintain the acceptable corrosion rate for N80 steel and CT. The authors also found that a 50–50 ratio of HEIDA and HCl at pH 2.5 required only 0.2% organic acid type inhibitor to reduce the corrosion rates by 10 times for N80, 13Cr, and CT. The lowered dose reduces the environmental footprint compared to the mineral acid fluid.

At pH 4, the corrosion rate of 20% HEDTA with 0.3% organic acid compatible corrosion inhibitor at 177 °C (350 °F) over 4 hours was found to be 13.6 mmpy (0.01 lb ft^−2^) on 13Cr and N-80.^34,48^

Hur *et al.*^63^ tested the corrosivity of 10 and 20% EDTA (pH = 7) on carbon steels SA 508 Cl.3, SA 516 Gr.70, and SA 285 Gr.C during sludge dissolution at temperatures ranging from 93 (200) to 150 °C (302 °F) and found acceptable corrosion rates in presence of 1% corrosion inhibitor. EDTA was evaluated because it does not tend to initiate new faults or propagate existing faults in steam generators during the chemical cleaning process. They noted that the corrosion rates increased linearly with cleaning time and the average corrosion at 150 °C (302 °F) was two times higher than at 130 °C (266 °F).

The corrosiveness of EDTA on P-110 was tested by Calderon *et al.*^55^ who showed that 10% disodium EDTA was more corrosive than 20% tetrasodium EDTA under different hydrodynamic regimes and temperatures. The cathodic depolarization effect was more pronounced in the case of the disodium EDTA. The addition of a mutual solvent, butylene–glycol, and a polyethoxylated nonionic surfactant did not change the corrosion rates of disodium EDTA. However, these additives increased the corrosion rates of tetrasodium EDTA by an order of magnitude.

20 wt% GLDA at pH 3.8 on L-80 at 149 °C (300 °F) over 6 h showed corrosion rates above the industry standard of 45.4 mmpy (0.05 lb ft^−2^) and thus required the addition of thiourea based corrosion inhibitors to lower it to an acceptable level.^64^ In the presence of 0.005% of corrosion inhibitor, the corrosion rate of GLDA on L-80 was found to be drastically reduced and be acceptable for oilfield applications. The authors also found that the thiourea-based corrosion inhibitor performed better than a quaternary-based corrosion inhibitor. Both these corrosion inhibitors at the same dosage were not sufficient to keep 20 wt% HEDTA (pH = 3.8) below the corrosion limits. C-95, Q-125, J-55, and P-110 required 0.1 vol% of the thiourea-based corrosion inhibitor for corrosion protection at 149 °C (300 °F) with 20 wt% GLDA.

Ng *et al.*^56^ noted high corrosion rates of GLDA, EDTA, HEDTA, and MGDA at pH 4 and temperatures of 149 (300 °F) and 177 °C (350 °F) for over 12 h of testing. However, with the addition of 1% corrosion inhibitor containing sulfur, these corrosion rates were brought down significantly. The authors delineated the corrosion mechanisms into (a) acid attack, (b) chelator enhanced dissolution, and (c) cathodic reduction. EDTA and GLDA had higher chelator enhanced corrosion rates than HEDTA and MGDA. EDTA and HEDTA had the highest cathodic reduction corrosion rates.

Corrosion tests conducted on L-80 and 13Cr using GLDA, ASDA, MGDA, and HEIDA at 149 °C (300 °F), pH 3.8, and 1000 psi showed unacceptable levels of corrosion for L-80 for all ligands, while 13Cr showed low corrosion only with GLDA.^21^ The corrosion rates of 20 wt% GLDA on L-80 between 93 (200 °F) to 204 °C (400 °F) were found to increase substantially with temperature but could be reduced to acceptable levels with the addition of 0.001% corrosion inhibitor containing alkoxylated fatty amines, alkoxylated organic acid, and thiourea.^65^ These rates were also found to be much lower for 13Cr and duplex at the same conditions. The authors concluded that GLDA was generally the most versatile environmentally friendly chelating agent in terms of corrosion, functionality in matrix acidizing, descaling, impact on tubular, completion, and environment.

Abdelgawad *et al.*^66^ studied the corrosivity of DTPA without a corrosion inhibitor at pH 12 and 120 °C (249 °F) on coiled tubing and determined the corrosion rate to be 6.4 mmpy (0.007 lb ft^−2^). They recommended using the DTPA-seawater system to eliminate excessive costs related to corrosion inhibitors and intensifiers, which could exceed 50% of the total costs in deep gas wells.

De Wolf *et al.*^62^ examined the corrosion rates of 20 wt% GLDA (pH = 3.8) on nickel-based alloys, Inconel-625 and Incoloy-925, at 177 °C (350 °F) over 6 h. The corrosion rate in the Inconel-625 and Incoloy-925 was found to be 10.7 (0.0118) and 3.6 mmpy (0.0040 lb ft^−2^), respectively. There were no signs of pitting on the surface of the coupons. Under North Sea conditions (10 mol% H_2_S, 5 mol% CO_2_, 121 °C (250 °F), 20 wt% GLDA (pH = 3.8) with 0.05% corrosion inhibitor containing polymeric ester quat and butyl diglycol (approved for use in the North Sea), resulted in acceptable corrosion rates. This formulation met all the Oslo Paris Convention for the Protection of Marine Environment of the Northeast Atlantic (OSPAR) requirements.

GLDA was also shown to have low corrosion rates with 22Cr and 13Cr at 150 °C (302 °F) without the use of corrosion inhibitors but HEDTA was found to be corrosive at similar conditions.^68^ Lal^69^ conducted tests using GLDA and EDTA at 149 (300 °F) and 177 °C (350 °F) at a pH of 4 on S13Cr-110 metal coupons and found the corrosion rates to be 7.5 (0.00827) and 0.1 mmpy (0.000114 lb ft^−2^), respectively.

Reyes *et al.*^70^ tested a GLDA/HF blend and showed acceptable corrosion rates on coiled tubing and drill pipe at temperatures below 160 °C (320 °F), but corrosion inhibitors were required at higher temperatures. The corrosivity of 25 wt% GLDA on L-80 at 127 °C (260 °F) in the presence of sour gas (7 mol%) and carbon dioxide (3 mol%) was examined by Nasr-El-Din *et al.*^71^ and found to require 1% corrosion inhibitor containing alkoxylated fatty amines, alkoxylated organic acid, and thiourea *N*,*N*′ dibutyl since the corrosion rate was too high (152.5 mmpy (0.168 lb ft^−2^)) without it. The iron and manganese concentration was reduced by 97 and 73% during the corrosion tests when the corrosion inhibitor was used. Nasr-El-Din *et al.*^72^ tested the corrosiveness of GLDA on L-80, Alloy 28, and Incoloy 925 under sour conditions at 300 °F (149 °C) for 6 hours and found the corrosion rates to be 9.7 (0.0107), 0.2 (0.0002), and 0.09 mmpy (0.0001 lb ft^−2^), respectively. In the presence of acidic gases such as H_2_S and CO_2_ at 121 °C (250 °F), a corrosion inhibitor was required for 20 wt% GLDA, resulting in low corrosion rates with L-80, 13Cr, duplex-2205, and alloy 28.^49^Tables 1 and 2 show a summary of the corrosion test results.

**Table 1:** Low-carbon steel corrosion rate summary

Metal	APCA	Concentration, wt%	*T*, °C	Inhibitor, v%	pH	Duration, hours	H_2_S/CO_2_	Corrosion rate, mmpy (lb ft^−2^)	Source
N-80	HEIDA	50	149	0.200	2.5	6	No	3.0 (0.0033)	14
	HEIDA	75	149	0.200	4.0	6	No	1.1 (0.0012)	
	EDTA	20	177	0	12.0	4	No	13.6 (0.0100)	48
	HEDTA	20	177	0.300	4.0	4	No	13.6 (0.0100)	
	GLDA	20	149	0	4	12	No	328.8 (0.724)	56
	HEDTA	20	149	0	4	12	No	364.6 (0.803)	
	EDTA	20	149	0	4	12	No	389.6 (0.858)	
	MGDA	20	149	0	4	12	No	291.5 (0.642)	
	GLDA	20	177	0	4	12	No	342.4 (0.754)	
	HEDTA	20	177	0	4	12	No	442.3 (0.974)	
	EDTA	20	177	0	4	12	No	485.9 (1.07)	
	MGDA	20	177	0	4	12	No	345.1 (0.76)	
	HEDTA	20	177	1	4	12	No	4.6 (0.0102)	
	MGDA	20	177	1	4	12	No	2.5 (0.00561)	
CT	HEIDA	50	149	0.200	2.5	6	No	1.8 (0.0020)	14
	HEIDA	75	149	0.200	4.0	6	No	0.9 (0.0010)	
	DTPA	15	120	0	12	6	No	6.4 (0.007)	66
L-80	GLDA	20	121	0	3.8	6	Yes	193.3 (0.2128)	42
	GLDA	20	121	0.050	3.8	6	Yes	3.5 (0.0038)	
	GLDA	25	127	0	Not mentioned	6	Yes	152.6 (0.1680)	71
	GLDA	25	127	1.000	Not mentioned	6	Yes	4.0 (0.0044)	
	GLDA	20	149	0	3.8	6	No	539.2 (0.5937)	64
	GLDA	20	149	0.001	3.8	6	No	512.8 (0.5647)	
	GLDA	20	149	0.005	3.8	6	No	23.8 (0.0262)	
	GLDA	20	149	0.001	3.8	6	No	35.8 (0.0394)	21
	GLDA	20	149	0	3.8	6	No	177.6 (0.1956)	
	GLDA	20	149	0	3.8	6	Yes	9.7 (0.0107)	72
	GLDA	20	149	0.001	3.8	6	No	<45.4 (<0.05)	65
	HEDTA	20	149	0	3.8	6	No	757.5 (0.8341)	64
	HEDTA	20	149	0.001	3.8	6	No	570.2 (0.6279)	
	HEDTA	20	149	0.005	3.8	6	No	118.1 (0.1300)	
	HEIDA	20	149	0	3.8	6	No	592.0 (0.6519)	21
	MGDA	20	149	0	3.8	6	No	417.4 (0.4596)	
	ASDA	20	149	0	3.8	6	No	628.1 (0.6916)	
P-110	EDTA	10	80	0	6.0	6	No	170.7 (0.1880)	55
	EDTA	20	80	0	10.0	6	No	0.09 (0.0001)	

**Table 2:** CRA corrosion rate summary

Metal	APCA	Concentration, wt%	*T*, °C	Inhibitor, %	pH	Duration, hours	H_2_S/CO_2_	Corrosion rate, mmpy (lb ft^−2^)	Source
13Cr	GLDA	20	121	0	3.8	6	Yes	334.1 (0.3679)	49
	GLDA	20	121	0.05	3.8	6	Yes	14.3 (0.0158)	
	GLDA	20	149	0	3.8	6	No	0.8 (0.0009)	21
	GLDA	20	149	0	3.8	6	No	7.3 (0.0080)	67
	HEDTA	20	149	0	3.8	6	No	300.6 (0.3310)	
	HEDTA	20	149	0	3.8	6	No	477.1 (0.5253)	49
	HEIDA	50	149	0.2	2.5	6	No	4.5 (0.0050)	14
	HEIDA	75	149	0.2	4	4	No	2.7 (0.0030)	
	HEIDA	20	149	0	3.8	6	No	53.6 (0.0590)	21
	MGDA	20	149	0	3.8	6	No	81.7 (0.0900)	
	ASDA	20	149	0	3.8	6	No	51.1 (0.0563)	
	HEDTA	20	177	0.3	4	4	No	9.1 (0.0100)	48
	EDTA	20	177	0	12	6	No	0.0000	
	GLDA	20	177	0.5	3.8	6	No	45.0 (0.0496)	49
	GLDA	20	177	0	3.8	6	No	315.9 (0.3478)	
	GLDA	20	150	0	—	6	No	7.3 (0.008)	68
	HEDTA	20	150	0	—	6	No	300.6 (0.3310)	
S13Cr	GLDA	20	177	0	3.8	6	No	17.0 (0.0187)	49
Alloy 28	GLDA	20	149	0	3.8	6	Yes	0.2 (0.0002)	72
22Cr	GLDA	20	150	0	—	6	No	0.09 (0.0001)	68
Incoloy 925	GLDA	20	149	0	3.8	6	Yes	0.09 (0.0001)	72
	GLDA	20	177	0	3.8	6	No	3.6 (0.0040)	49
Inconel-625	GLDA	20	177	0	3.8	6	No	10.7 (0.0118)	49
Duplex	HEDTA	20	177	0	3.8	6	No	0.0000	49
	GLDA	20	177	0	3.8	6	No	0.09 (0.0001)	

Due to corrosion mechanisms of chelating agents, corrosion may be inhibited by adding corrosion inhibitors that act by blocking adsorption sites or cathodic sites. Campbell and Eick^73^ showed the inhibition of goethite, an iron oxohydroxide (α-FeO(OH)), from EDTA dissolution using phosphates, molybdates, and selenite. These oxyanions compete with EDTA for adsorption sites on the mineral surface, preventing EDTA adsorption and dissolution of the rock. However, they also show that insufficient inhibitor concentration can result in incomplete coverage of adsorption sites on the surface, resulting in EDTA adsorption to an extent comparable to the absence of inhibitors at pH > 6. They also noted an increase in goethite dissolution, which they attributed to EDTA being forced into a dissolution enhancing mononuclear conformation to adsorb to the remaining sites. This dissolution study could be used to deal with corrosion issues as well.

Shi *et al.*^74^ prepared GLDA (pH = 4) by diluting a high pH stock solution and changing its pH using acetic acid and found that it exhibited a better acidification effect on Baota limestone than a solution prepared with HCl as the pH modifier. 5 wt% GLDA without any corrosion inhibitor showed 24 h corrosion rates of 0.9 (0.004) and 15.9 mmpy (0.07 lb ft^−2^) at 25 (77 °F) and 80 °C (176 °C), respectively. They postulated that physical and chemical adsorption decreased the interactions of H^+^ ions with the metal and thus reduced the corrosion rates compared to standalone HCl or acetic acid. A new sulfate scale dissolver composed of ethylenediamine, NTA, EDTA, chloroacetic acid, and a surfactant with a pH of 12.5 showed a corrosion rate of 12.7 mmpy (0.014 lb ft^−2^) at 90 °C (194 °F) and 6 h of testing.^75^ A 0.4 M DTPA in presence of 1 vol% corrosion inhibitor and mutual solvent yielded a corrosion rate of 0.9 mmpy (0.001 lb ft^−2^) (N80) during an iron sulfide scale treatment process at 65 °C (150 °F).^76^ Luo *et al.* studied a DTPA–sodium polyacrylate blend (pH = 12) for the dissolution of barite and evaluated the blend's corrosion performance at 90 °C (194 °F).^77^ The blend showed negligible corrosion rates for N80 and 13Cr for a testing time of 10 h. Disodium EDTA solutions can corrode carbon steel as high as 16.6 mm per year, which is considered high for engineering operations.^78^

## APCAs as corrosion inhibitors

Chelating agents are only corrosive if the chelated product is soluble. Therefore, if the ligand can form an insoluble chelated product after binding to the surface site on the metal, it can act as a corrosion inhibitor.^79^ Chelating agents may also inhibit corrosion if they form bi- or multinuclear complexes on the surface site, as it is energetically unfavorable to remove these complexes.^80^

EDTA has been examined thoroughly by several authors as an inhibitor for various types of metals in acid. It has been shown to inhibit corrosion on mild steel and aluminum in 0.5 M HCl through adsorption of the EDTA molecule on the metal surface.^81,82^ Both authors demonstrate that EDTA was able to both inhibit corrosion by acting as a mixed inhibitor and prevent pitting on the metal surface immersed in the acid solution. Even in 1 M of HCl, EDTA was still able to perform comparatively well against thiourea, a conventional corrosion inhibitor at room temperature.^83^ Zor *et al.*^84^ showed that aluminum surfaces could be protected from chloride corrosion by EDTA and that this inhibition was optimal at pH 9 with low concentrations of EDTA (10^−4^ M) at room temperature.

Inhibition properties of EDTA can be further enhanced through the addition of other ions and inhibitors. Zinc(ii) ions (Zn^2+^) and trisodium phosphate (Na_3_PO_4_) have been shown to exhibit synergistic effects when used with EDTA to inhibit corrosion.^85^ The authors show that at the optimal pH of 7, 98% inhibitor efficiency is obtained due to the formation of an insoluble layer of ferrous EDTA, ferrous Na_3_PO_4_, and zinc(ii) hydroxide. Another set of additives that have been shown to enhance the inhibition properties of EDTA is hydroxylamine sulfate and Fe^2+^.^86^ Finally, the inhibiting properties of EDTA on steel surfaces can be strengthened through the addition of Fe^3+^ ions to the solution.^81^

A magnesium hydroxide-EDTA coating reduced the corrosion rate of bare AZ31 Mg alloys by one or two orders of magnitude.^87^ EDTA accelerated the formation of the protective coating.

## APCAs degradation

Understanding the degradability of APCAs is an important factor when designing treatments. The effects of chelating agents on the environment have been comprehensively studied over the past few decades. Because of their ability to chelate metal ions, chelating agents in the environment can influence the speciation and bioavailability of metals, as well as remobilize toxic heavy metals into drinking and groundwater from sediments and aquifers.^88,89^

APCAs have also been shown to harm certain types of bacteria by destroying cell membranes and harm plants by increasing toxic heavy metal uptake.^90^ Furthermore, APCAs may cause water eutrophication due to the presence of nitrogen atoms in their structure that results in undesired algae blooms.^90,91^ In such cases, these effects can be minimized if the chelating agent readily degrades when introduced to the environment.

APCAs have been used in the oil and gas industry for several applications including oilfield stimulation, iron control, scale removal, and enhanced oil recovery. The effectiveness of such treatments depends on the performance of the chemical agents. Its degradation under extreme conditions can render the treatment ineffective. Degradation of APCAs can be divided into three broad degradation processes: thermal, photo-, and biodegradation. Due to the frequent use of APCAs in the oil and gas industry, it is critical to understand the level of resistance of ligands to these types of degradation. Understanding the limitations of each type of APCA can help researchers and industry professionals to design an effective downhole treatment. Also, knowing the degradation products of chelating agents, especially those from thermal decomposition, allows for the identification of problems that may arise from negative interactions of these products with other additives in the solution. Some APCAs degrade to yield other APCAs of lower stability constants, which may still be able to carry out the function of the parent APCA. The resulting lower stability may lessen the impact of degradation on chelating agent systems.

### Thermal degradation of APCAs

When organic compounds are used in the oilfield, thermal stability becomes an important consideration due to harsh downhole conditions. These temperature limitations are usually indicative of the operational limit of the chemicals used, and APCAs are no exception. Therefore, it is important to determine the thermal stability of these APCAs before they are used in the field. It is also important to examine the degradation products of these molecules to determine if they will precipitate or participate in undesired side reactions with other additives.

The thermal stability of chelating agents, their various thermal degradation pathways, and the effect of various environmental factors on the degradation products and process have been thoroughly studied. Martell *et al.*^92^ studied the thermal degradation of both NTA and EDTA and showed that at 260 °C (500 °F) and pH 9.5, EDTA hydrolyzes to its primary degradation products HEIDA and IDA in half an hour. HEIDA was later shown to undergo further hydrolysis to yield ethylene glycol and IDA. Eventually, the primary degradation products were determined to be substituted methylamines.

NTA decomposed at 293 °C (560 °F) in a stepwise, non-hydrolytic decarboxylation process, starting from the initial degradation product, *N*-methyliminodiacetic acid (MIDA), to methylsarcosine, and finally yielding trimethylamine. At low pH, however, degradation products of NTA were found to be IDA, MIDA, sarcosine, glycine, and *N*,*N*-dimethylglycine with the eventual degradation products being carbon monoxide (CO), carbon dioxide (CO_2_), formaldehyde, and methylamines.^93^ However, these decompositions are not common since typical temperatures in the field are far below these levels.

**Fig. 4:**
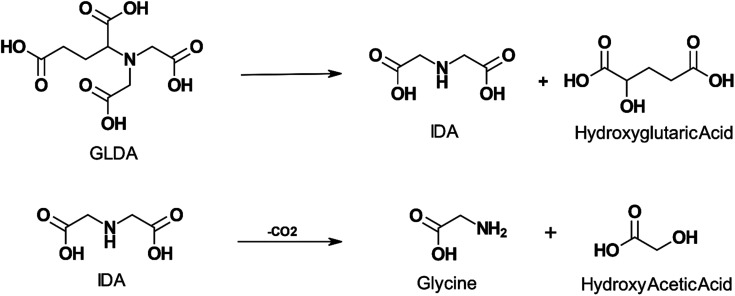
Proposed mechanism for GLDA degradation at pH 4 and exposure to temperature up to 200 °C for a period above 4 hours (adapted from ref. 96).

The thermal decomposition of GLDA was studied by several authors.^20,21,94–96^ GLDA exhibited similar thermal stability to HEDTA when heated for 4 hours at 149 °C (300 °F) and 177 °C (350 °F) and the decomposition products were cyclic GLDA and formic acid.^20^ Sokhanvarian^95^ examined the decomposition of GLDA at the same temperatures and a pH of 4 up to 12 hours. The GLDA decomposition products were identified through mass spectrometry as monosodium glutamate-monohydrate, IDA, oxotetrahydrofuran-2 carboxylic acid, hydroxyglutaric acid, and acetic acid. IDA and hydroxyglutaric acid were identified as the primary degradation products. The authors also suggest a mechanism for the thermal decomposition of GLDA at low pH in Fig. 4. MGDA and HEIDA were extremely stable at temperatures up to 177 °C (350 °F), experiencing no degradation over 6 hours.^21^ However, ASDA was shown to degrade easily, with less than 10% of the APCA remaining when degraded at 149 °C (300 °F) for the same duration. This would require the application of an APCA with adequate thermal stability for high-temperature wells since the degradation products will no longer be able to perform the function of the original ligand, such as iron control, scale removal, or matrix acidizing.

#### Factors influencing thermal degradation

By testing the degradation products of EDTA at various pH values at 200 °C (392 °F), Venezky and Moniz^97^ showed that a decrease in pH results in a corresponding decrease in thermal stability and that pH also influences the stepwise degradation of EDTA. The improved stability of high pH solutions of EDTA was postulated to be because of higher resonance stabilization of the free carboxylate ions. Also, the degradation process is faster in presence of H^+^ ions.

**Fig. 5:**
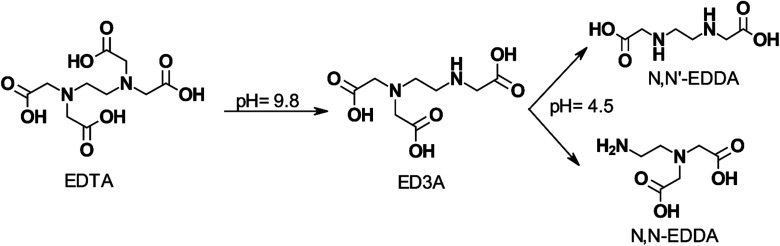
Proposed effect of pH on the stepwise degradation of EDTA at 200 °C (392 °F) (adapted from ref. 97).

Fig. 5 shows the effect of pH on EDTA degradation at 200 °C (392 °F). EDTA is postulated to lose one –CH_2_COOH group and degrade to ethylenediaminetriacetic acid (ED3A) which undergoes further decomposition as the pH is lowered.

**Fig. 6:**
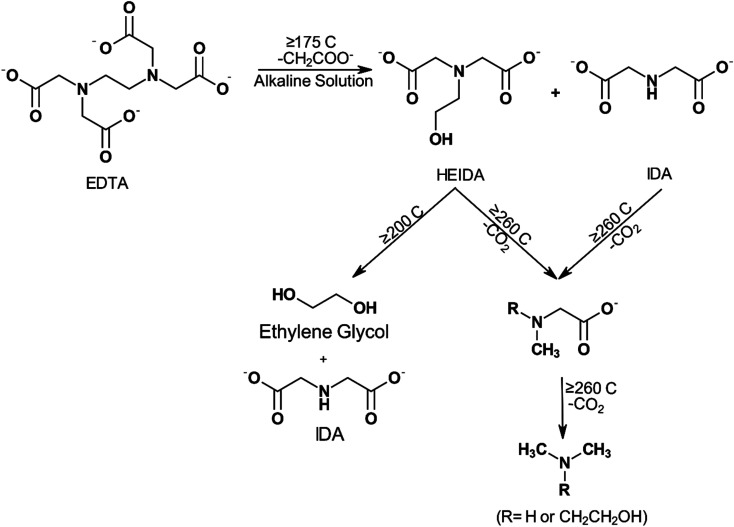
Proposed schematic of EDTA degradation and the effects of temperature on the final products (adapted from ref. 104).

Increasing H^+^ ion concentration resulted in higher concentrations of MIDA when NTA is thermally decomposed.^93^ Dillon^98^ degraded EDTA at pH 7 and 250 °C (482 °F) for a week and found that only methyl and ethyl substituted amines remained. Boles *et al.*^99^ evaluated disodium EDTA degradation at pH 4.6 and temperatures of 125 (257 °F), 175 (347 °F), and 200 °C (392 °F). These researchers concluded that the resulting primary degradation product was MIDA instead of HEIDA or IDA. They found that the half-life of disodium EDTA was 1.4 ± 0.4 hours at 200 °C (392 °F). Below 1 week of degradation, the primary degradation products retained the carboxyl functional groups capable of binding metal ions. However, its efficiency in chelating transition and actinide metals reduced by 6 to 22 orders of magnitude at 25 °C (77 °F) compared to EDTA. Beyond 1 week of degradation, the EDTA was decarboxylated into methyl and ethyl substituted amines, thereby removing any metal chelation abilities. The presence of dissolved oxygen also influences the rate of degradation, where higher oxygen content leads to higher thermal degradation. During mixing or pumping, oxygen will inadvertently be added to the system. Dissolved oxygen (O_2_) concentration was also found to affect the rate of decomposition of EDTA, with higher concentrations of O_2_, resulting in a higher rate of decomposition.^100^ In the presence of 5% palladium on carbon catalyst, NTA can be degraded by O_2_ at 90 °C (194 °F) to yield IDA, carbon dioxide, water, and oxalic acid.^101^ Thermal degradation of EDTA was found to decrease the amount of free EDTA rapidly at higher temperatures.^102^ Final degradation products of EDTA have also been shown to be dependent on the temperature of the system^92,103^ (Fig. 6).

The effects of metal ions on the thermal degradation of chelating agents have also been examined. This is important in applications such as scale removal and iron control since thermal degradation of the metal chelate would release the ion held back into solution with undesirable consequences. In general, APCA chelates are more stable than their protonated forms. Motekaitis *et al.*^35^ tested the effects of various divalent cations on the thermal stability of EDTA at pH 10.4 and found an inverse correlation between the thermal stability of the metal chelate and the stability constant between EDTA and the metal ion. This is due to the predominant mechanism of dissociation followed by degradation at high temperatures. The higher stability complexes dissociate slowly and lead to better thermal stability than the less stable complexes. The presence of phosphate or silicate ions accelerated the degradation process of the Ca(ii)–EDTA complex product. The thermal stability of NTA with several divalent cations and Fe^3+^ was tested at 300 °C (572 °F) by Booy and Swaddle.^105^ They showed that NTA^3−^ was more stable than several NTA chelates such as Fe(ii)–NTA, Fe(iii)–NTA, and Cu(ii)–NTA. However, Co(ii)–NTA was demonstrated more thermal stability than NTA^3−^.

HEDTA and GLDA were shown to be stabilized by the addition of various salts commonly used in drilling or clay stabilization, including cesium formate, potassium chloride, and ammonium chloride.^95^ It was also found that aminopolycarboxylic acids with a higher number of nitrogen atoms are generally more thermally stable. Calcium ions (Ca^2+^), which are common during limestone acidizing, were also found to stabilize GLDA.^96^ Resonance stabilization of free carboxylate groups is thought to provide increased thermal stability in the presence of cations and higher pH.^95,97^ Hydrated sodium salts of EDTA were found to be less stable at a much lower temperature than their aqueous form.^106^

Redox reactions with metal ions that eliminate APCA molecules have also been observed. Ethylenediaminetriacetic acid (ED3A), IDA, and HEIDA were found to be the degradation products of EDTA, while NTA yielded IDA as the major product. Fe^3+^ was observed to be reduced to Fe^2+^ and Cu^2+^ to copper metal (Cu^0^). Booy and Swaddle^105^ also decomposed NTA–Fe^3+^ and NTA–Cu^2+^ chelates and made similar observations with the former yielding IDA, sarcosine, and dimethylamine, with the latter producing IDA, formaldehyde, and CO_2_. Only 2.5% of NTA was degraded after 6 hours of exposure to 160 °C (320 °F). However, at 220 °C (428 °F), the rate of degradation was faster and about 75% of NTA was degraded after 8 hours of exposure.^107^ Using thermogravimetric and diffusal thermal analysis (TG/DTA), ED3A was proven to be the major component of EDTA oxidation by Fe^3+^.^108^ Lambert and Mason^109^ reacted hexacyanoferrate with EDTA at 50 °C (122 °F) and observed Fe reduction. Studies of the reaction kinetics and mechanism of EDTA, DTPA, NTA, and CDTA with cerium(iv) ions (Ce^4+^) in perchloric acid media at room temperature show a reduction of Ce^4+^ to cerium(iii) ions (Ce^3+^).^110–112^ Hanna *et al.*^112^ suggest the degradation products of EDTA to be ethylenediamine and glycine when oxidized by Ce^4+^ and propose a mechanism for their formation. Therefore, more chelating agents would be required to adequately control iron or remove iron-based scale.

### Biodegradation

In recent times, the oil and gas industry has placed an ever-growing emphasis on the use of environmentally friendly materials, ranging from demulsifiers to drilling fluids.^160^ The biodegradability of chelating agents is an important factor when selecting which type of APCAs to use, especially in offshore environments. Furthermore, selecting more biodegradable ligands that can be degraded with bulk waste may be preferable to those that do not and therefore require an extra layer of treatment to remove.

Industrial use of chelating agents has rendered its disposal into natural waters. Biodegradation of organic compounds is the primary method to remove them from the environment.^113^ Chelating agents in the environment can influence the speciation and bioavailability of metals, as well as remobilize toxic heavy metals into drinking and groundwater from sediments and aquifers.^88,89^ They may also cause water eutrophication due to the presence of nitrogen atoms in their structure.^36,90^ In general, the biodegradability of chelators in the oilfield is evaluated using OECD (Organization of Economic Cooperation and Development) tests and standards. When undergoing biodegradability tests, biodegradable chelating agents, such as *S*,*S*-EDDS, are often tested alongside as a control to determine if the conditions of the test are favorable for biodegradation.

**Fig. 7:**
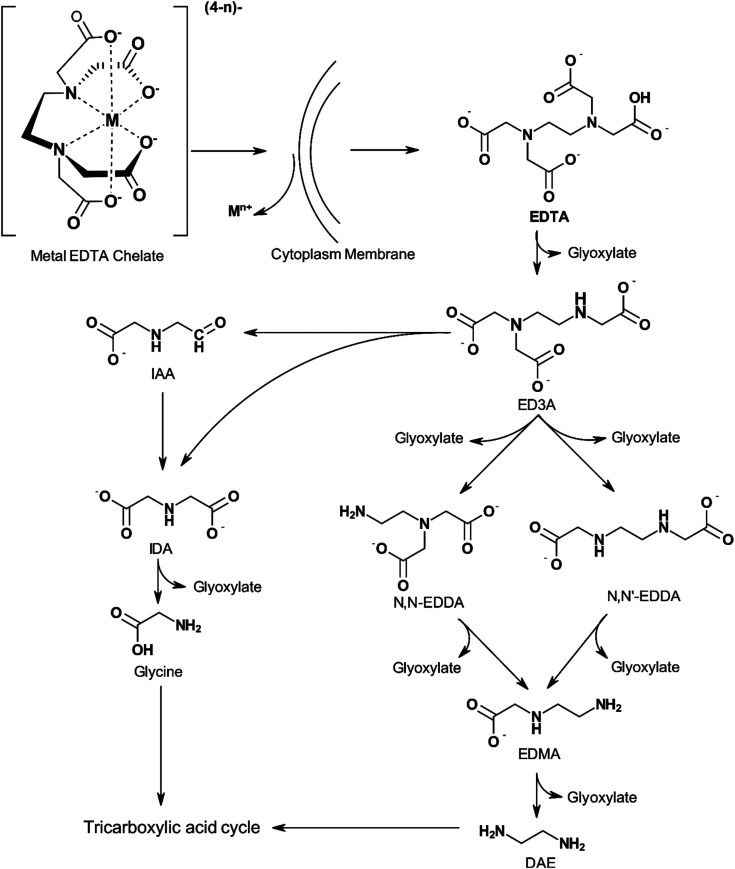
Proposed biodegradation pathway of EDTA by DSM 9103 (adapted from ref. 124).

The biodegradability of APCAs is influenced by the number and character of nitrogen atoms present in the molecule. Chelators containing a single nitrogen atom, such as NTA, are biodegradable, whereas those containing two or more nitrogen atoms, such as EDTA and DTPA, cannot be degraded by typical assays.^13,24,114^ Means *et al.*^115^ showed that NTA is more biodegradable than EDTA and DTPA in the long term. They also observed that the rates of degradation of the three APCAs were not high enough to preclude concern about their release to the environment. Biodegradation of NTA was found to occur in wastewater, river water, and using the activated sludge process, evolving CO_2_, water, and inorganic nitrogen as degradation products.^116^ EDTA, however, can be biodegraded by certain strains of bacteria found in aerated lagoons, secondary wastewater treatment facilities, industrial sewage, and lab-grown sources.^24,36,117–122^Fig. 7 shows a pathway for biodegradation of EDTA. ED3A and IDA are the main metabolites of EDTA oxidation by highly concentrated biomass from an aerated lagoon receiving EDTA-containing wastewaters. Other products such as EDDA, ethlyenediaminemonoacetate (EDMA), NTA, and glycine were detected in low concentrations. The type of nitrogen atom, secondary or tertiary, also appears to play a role in the stability of the chelating agent as shown by the higher susceptibility of ethylenediaminediacetic acid (EDDA) to degradation than EDTA.^24,123^

The substituents and stability constants of the metal chelate also affect its biodegradability. The effect of various substituents in ethylenediamine derivatives was investigated by Sykora *et al.*^24^ They found that biodegradability increased in the order –COCH_3_, –CH_3_,–C_2_H_5_, –CH_2_CH_2_OH, –CH_2_COOH. These authors also showed that EDTA, HEDTA, and DTPA were not degraded by prolonged exposure to activated sludge. The lowering of the degree of substitution helps in making the compound more biodegradable. For example, lowering the number of acetic acid groups in EDTA from four to two, hence making it EDDA, increases the biodegradability of the compound.

Understanding the biodegradability of metal-chelate complexes is important because ligands are often present in the chelated form in waste solutions. Therefore, biodegradability test results of metal chelates can vary widely primarily depending on the cell's ability to deal with specific metal ions. Degradation dependence on stability constants for EDTA was only shown for whole cells and not for EDTA–monooxygenases.^125^

Metal–EDTA complexes with stability constants of 12 and lower, except for Zn–EDTA, could be degraded by the strain BNC1.^120^ This would also include Ca–EDTA, a common product when EDTA is used for matrix acidizing. The presence of these complexes was also shown to not influence the metabolization of uncomplexed EDTA or degradable metal–EDTA complexes. The degradation of EDTA is usually accompanied by the precipitation of metal salts, an increase in the flocculation of cells, and increasing pH. DSM 9103 was also tested on EDTA, DTPA, and some divalent EDTA metal complexes, though degradation of DTPA was only 65 to 70% of EDTA.^121^ However, using ultra-filtered cell-free extracts of BNC1 or purified EDTA–monooxygenases of DSM 9103 showed a great increase in biodegradation rates and an absence of dependency of stability constants.^125^ Satroutdinov *et al.*^126^ investigated *Pseudomonas* sp. LPM-410 and LPM-4 and showed improved degradation of some EDTA chelates compared to DSM 9103, but were unstable in cobalt (Co), Cu, and Pb complexes.

Microorganisms in soil cultures under aerobic conditions were also found to be able to degrade EDTA and some of its metal chelates.^117^ Madsen and Alexander^127^ investigated the effects of certain cations on the biodegradability of oxalate, citrate, NTA, and EDTA and found that EDTA and NTA chelates, except for NTA–Ca^2+^, were not biodegradable. However, at sufficiently low levels of NTA, a *Pseudomonas* species was found to degrade NTA chelates other than Ni chelates.^128^

Allard *et al.*^129^ examined the biodegradability of ^14^C EDTA and DTPA iron complexes with modified OECD tests and showed that they were non-biodegradable. Uncomplexed DTPA and its Fe^3+^ chelate were also found to be non-biodegradable by Metsärinne *et al.*^130^ and Alarcón *et al.*^131^ No microorganisms have been reported to be able to use DTPA as a sole source of carbon and energy.^125^

**Fig. 8:**
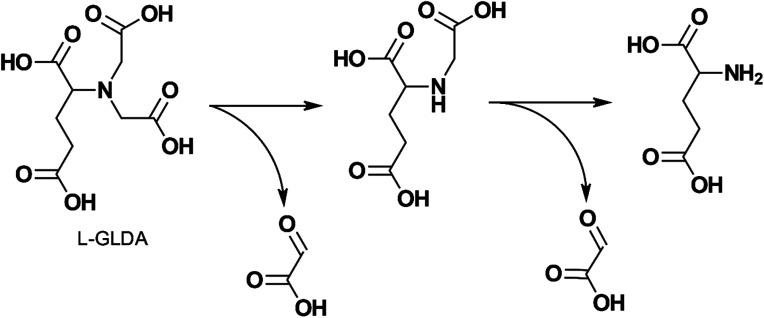
Proposed biodegradation pathway of l-GLDA by BG-1 (adapted from ref. 132).

The degradability of l-GLDA, D-GLDA, NTA, and several of their metal chelates were studied by Van Ginkel *et al.*^132^ with OECD biodegradation tests and an isolate strain BG-1 (also known as Rhizobium radiobacter). l-GLDA was found to be readily biodegradable in all tests and its chelates were readily degraded by BG-1. Fig. 8 shows the author's proposed degradation pathway. D-GLDA, an enantiomer of l-GLDA, did not degrade in activated sludge, but degraded in acclimatized sludge and the SCAS (Semi Continuous Activated Sludge) test at a slower rate than l-GLDA. Mahmoud *et al.*^94^ showed that GLDA was degraded by 60% using the OECD 301D test and completely degraded in the OECD 303A test, indicating it was biodegradable.

Similarly, Witschel and Egli^133^ tested the biodegradability of [*R*,*R*]-EDDS, [*R*,*S*]-EDDS, and [*S*,*S*]-EDDS and observed that [*R*,*R*]-EDDS was non-biodegradable, while [*S*,*S*]-EDDS was readily biodegradable. Chen *et al.*^134^ used computational and experimental methods to study the stability and degradation of *S*,*S*-EDDS, and EDTA chelates, and this showed that the biodegradation of both molecules involves preferential cleavage of the C–N bond. Stereo conformation was also shown to affect the degradability of ASDA with only L-ASDA being easily biodegradable.^135^

### Photodegradation

In addition to degradation by thermal and biological processes, chelating agents can also undergo photodegradation. Understanding the photo-degradability of chelating agents presents a third alternative to degrade chelating agents should biodegradation or thermal degradation prove ineffective. In addition, chelating agents susceptible to photodegradation would have to be stored appropriately in the field to prevent excessive degradation of the chemical.

Means *et al.*^115^ tested the photo-, bio-, and chemical degradation rates of NTA, DTPA, HEDTA, and EDTA over 173 days. EDTA and NTA were observed to be relatively resistant to photodegradation, while DTPA experienced high rates of degradation. HEDTA was observed to have degraded completely by the end of the experiment.

In the absence of oxygen, EDTA photodegradation occurs *via* decarboxylation, while the presence of O_2_ or hydrogen peroxide (H_2_O_2_) involves the cleavage of a C–N bond.^136^ Selieverstov *et al.*^137^ examined the effects of ultraviolet (UV) irradiation and H_2_O_2_ on the degradation of EDTA solutions under alkaline and acidic conditions. These researchers found that under acidic conditions (citric acid), EDTA was stabilized to photodegradation by citric acid but adding H_2_O_2_ allowed for efficient degradation. In alkaline conditions, degradation of EDTA was increased by using a combination of UV irradiation and H_2_O_2_. Unlike EDDS, the susceptibility of EDTA to photodegradation was shown to be dependent on the presence of metal ions.^138^

Since chelating agents generally exist as metal chelates post-treatment, understating the rate of their photodegradation is important when considering their environmental impact. This, along with biodegradation of metal chelates, can provide a good estimate of the lifespan of chelating agents in the environment, and can reveal if waste containing chelating agents requires treatment before it is discharged.

Ferric chelates are usually tested because they are the most common complex in water considering the concentration and stability constants of the ions present.^88,139^ Ferric EDTA was found to degrade in presence of fluorescent and incandescent lamps and a yellow-tan precipitate containing majorly iron was produced.^140^ Hill-Cottingham^141^ examined the photosensitivity of ferric EDTA, HEDTA, and DTPA and found them to be degradable in sunlight. Lockhart and Blakely^142^ carried out photodegradation tests of EDTA with various divalent and trivalent cations and concluded that EDTA chelates of Mn^2+^ and Fe^3+^ were the most degradable of the chelates tested. Sources of Mn^2+^ include corrosion of low-carbon steel while Fe^3+^ is typically obtained from hematite, pumped acid solutions, rust, and corrosion of tubulars.

The pH of the solution was shown to influence the degradability of the complex because higher pH values would result in the preferential formation of uncomplexable iron hydroxides.^138,142,143^ Degradation of ferric NTA and EDTA chelates at 30 °C (86 °F) and pH 9–10 were observed to involve decarboxylation of the chelating agent and reduction of ferric ions to their ferrous state.^143^ CO_2_, formaldehyde, and the Fe^2+^ complexes of NTA and IDA were observed for ferric NTA degradation, while CO, formaldehyde, Fe^2+^, and ED3A were formed from the degradation of ferric EDTA.^142,144^

**Fig. 9:**
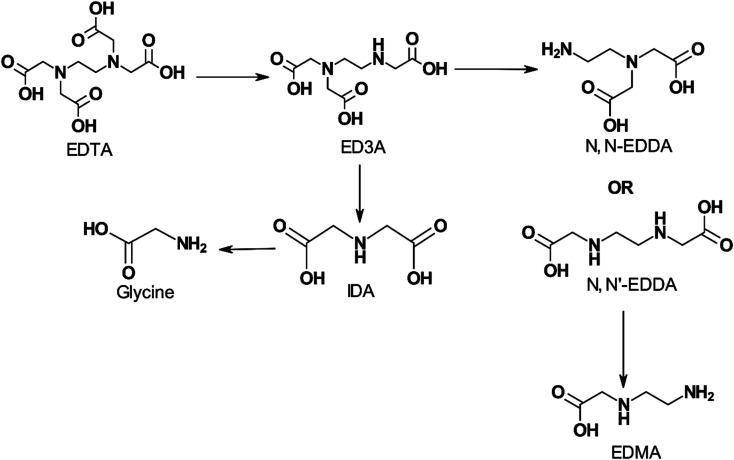
Proposed photochemical conversion of EDTA in aquatic conditions at neutral pH (adapted from ref. 139).

Svenson *et al.*^139^ degraded ferric chelates of NTA, DTPA, and EDTA at pH 7 and showed that NTA–Fe^3+^ was the most resistant to photodegradation, while DTPA–Fe^3+^ exhibited the shortest half-life. This work also proposed steps for the photochemical conversion of EDTA at neutral conditions but did not specify the byproducts of the reactions (Fig. 9).

Metsärinne *et al.*^130^ examined the photodegradation of DTPA in the presence and absence of Fe^3+^ in distilled water and lake water using sunlight and UV radiation at the range of 315–400 nm emitted by blacklight lamps and observed that free DTPA was more resistant to photodegradation than DTPA–Fe^3+^. They observed almost 90% degradation during the first two weeks of DTPA photodegradation. DTPA is said to form photolabile Fe(iii) complexes which lead to the rapid elimination of DTPA in lake water. Tests using IDA–Fe^3+^ showed photodegradability in acidic solution, though no degradation is observed at neutral conditions.^145^

## Field applications of APCAs

Conventional APCAs, such as EDTA, NTA, HEDTA, and DTPA, have been widely used in the oil and gas industry, including scale removal and acidizing treatments. However, these ligands present a danger to the environment due to their recalcitrance and toxicity. A new generation of biodegradable APCAs has generated increasing interest due to their ability to rival the traditional APCAs in terms of stability and performance. Such chelating agents include HEIDA, MGDA, GLDA, and ASDA. MGDA and GLDA are renowned for their ability to chelate calcium and magnesium ions.^31,146–148^

Zack *et al.*^148^ patented a system using MGDA in the presence of methane sulfonic acid to dissolve scales, such as calcium carbonate and showed effective dissolution and inhibition at 176 °F (80 °C). MGDA has also been designed for use as a stimulation fluid alongside HF by Pascarella and Reyes^149^ and as a self-diverting mixed acid system.^150^ MGDA, GLDA, and ASDA have also shown promise as stimulation and fracturing fluids.^151,152^ MGDA can also be used in ASP (Alkali Surfactant Polymer) solutions as a scale inhibitor.^153^

GLDA has also seen many successes when used in the field. GLDA was shown to improve the production of a sour gas carbonate reservoir by 110% after treatment,^72^ and that of a sandstone gas reservoir for three times the duration of conventional treatments.^71^ GLDA has also been used to successfully stimulate SAGD producing wells.^154^ HEIDA has also been shown to be an effective scale remover that can be used in place of EDTA.^14^ Sopngwi *et al.*^155^ used an APCA/HF (hydrofluoric acid) system to successfully stimulate a well in a sandstone reservoir in the Gulf of Mexico and showed an increase in the production rate of 30%, while the acid system showed a corrosion rate of 14.5 mmpy (0.016 lb ft^−2^) on 13Cr in the presence of 0.6% inhibitor. More recently, a similar system was applied to offshore wells in West Africa and showed an improvement of 48% to production rates.^156^ Panait *et al.*^157^ investigated the application of GLDA as a matrix acidizing fluid in Romanian heavy oil fields and observed an improvement in the performance of the wells. GLDA was also used at pH 10 as a scale remover to treat wells damaged by iron, calcium, and magnesium scale.^158^ Santos *et al.*^159^ used 20% GLDA at pH 3.8 to acidize offshore wells in the North Sea and showed notable improvement to the production rate. Wang *et al.*^161^ applied a combination of a chelating agent preflush and the main body of 15 wt% HCl to stimulate a gas well at 365 °F (185 °C). They showed an increase of 50 000 m^3^ per day in production rate following the treatment. Channa *et al.*^162^ acidized oil wells using 50 vol% GLDA solution and observed a 30 to 40% increase in productivity. Ting *et al.*^163^ used a chelating agent-based mud acid system to stimulate a reservoir of 49 °C (120 °F) and were able to increase production to approximately 200%.

## Conclusions

Chelating agents have a wide range of applications in the oil and gas industry that involve extended exposure to harsh conditions. It is important to understand what influences the temperature limits to ensure that the chelating agents are still active in these harsh oilfield conditions. In addition, corrosion caused by chelating agents has to remain within the industry's acceptable limit to minimize damage to the downhole equipment and therefore minimize expenditure. Understanding the operational limits as well as the degradability of chelating agents in the environment is essential for selecting the appropriate chelating agent when designing treatments.

The main conclusions of this review paper can be summarized in the following points:

(1) Acidic solutions of chelators are more corrosive compared to basic solutions of chelating agents and the corrosion rate of both cases increases as temperature increases.

(2) Low carbon steel corrosion by ligands occurs through a 2-step process: chelator enhanced dissolution of the iron oxide layer followed by a redox reaction between the base metal and the chelating agent.

(3) The corrosion due to chelating agents is much less with CRA tubulars compared to LCS but still requires corrosion inhibitor at temperatures above 300 °F (149 °C).

(4) Corrosion by chelating agents can be reduced by adding the appropriate corrosion inhibitors such as alkoxylated fatty amines.

(5) The thermal stability of deprotonated or complexed ligands was found to be higher than protonated chelating agents due to multiple factors including resonance stabilization and more stable chelate products.

(6) The presence of salts such as cesium formate, potassium chloride, and ammonium chloride increases thermal stability in chelators.

(7) Lower pH conditions or the presence of oxygen reduces the thermal stability of chelating agents.

(8) The ligands' biodegradability is influenced by several factors including the number of nitrogen atoms and the complexity of their chemical structure.

This work provides the petroleum industry with an adequate and informative summary of factors contributing to the corrosion of chelating agents and their susceptibility to various types of degradation. The knowledge gained through this review can help readers determine a suitable chelating agent for specific applications and avoid major pitfalls. It can also help future researchers in identifying gaps in literature where additional research is needed. One such area is identifying degradation behavior mechanisms for other chelating agents not studied in this paper.

## Conflicts of interest

There are no conflicts to declare.

## Abbreviation

APCA: Aminopolycarboxylic acid
ASDA: l-Aspartic acid *N*,*N*-diacetic acid
CDTA: *Trans*-l,2-cyclohexylenediaminetetraacetic acid
CRA: Corrosion resistant alloys
DAE: Diaminoethane
DT3A: Diethylenetriaminetriacetic acid
DT4A: Diethylenetriaminetetraacetic acid
DTPA: Diethylenetriaminepentaacetic acid
ED3A: Ethylenediaminetriacetic acid
EDDS: Ethylenediaminedisuccinic acid
EDMA: Ethylenediaminemonoacetic acid
EDTA: Ethylenediaminetetraacetic acid
HCl: Hydrochloric acid
HEDTA: Hydroxyethyl ethylenediaminetriacetic acid
HEIDA: Hydroxyethyliminodiacetic acid
HF: Hydrofluoric acid
IAA: Iminoacetaldehydeacetate
ICP: Inductively coupled plasma
IDA: Iminodiacetic acid
l-GLDA: l-Glutamic acid *N*,*N*-diacetic acid
LCS: Low carbon steel
MGDA: Methylglycinediacetic acid
MIDA: *N*-Methyliminodiacetic acid
MS: Mass spectrometry
MSG: Monosodium glutamate
*N*,*N*′-EDDA: *N*,*N*′-Ethylenediaminediacetic acid
*N*,*N*-EDDA: *N*,*N*-Ethylenediaminediacetic acid
NMR: Nuclear magnetic resonance
NTA: Nitrilotriacetic acid
*o*-PDA: 1,2-Phenylenediamine

## Supplementary Material
